# Seasonal variability of prevalence and occurrence of multiple infections shape the population structure of *Crithidia bombi*, an intestinal parasite of bumblebees (*Bombus* spp.)

**DOI:** 10.1002/mbo3.35

**Published:** 2012-09-23

**Authors:** Mario Popp, Silvio Erler, H Michael G Lattorff

**Affiliations:** Institut für Biologie, Molekulare Ökologie, Martin-Luther-Universität Halle-WittenbergHoher Weg 4, 06099, Halle (Saale), Germany

**Keywords:** Effective population size, heterozygosity, host– parasite interaction, intensity of infection, multiple infections

## Abstract

Ergonomic growth phases of annual social insect societies strongly influence horizontally transmitted parasites. Herein, we focused on the impact of temporal changes in host demography on the population structure of a horizontally transmitted parasite. Seasonal fluctuations in prevalence and the occurrence of multiple infections of the gut parasite *Crithidia bombi* were analyzed in repeatedly sampled populations of two common bumblebee (*Bombus* spp.) species. Prevalence of *C. bombi* was greatest in the middle of the foraging season and coincided with the maximal occurrence of multiple infections. Both decline later in the season. The genetic structure of the parasite population also showed strong seasonal fluctuations with a drastic decline in effective population size and an increase in linkage disequilibrium when infection rates were highest. These effects are mainly attributable to significant changes in parasite allele frequencies leading to selection of specific alleles and increasing the frequency of homozygote genotypes in the middle of the season. Within host, competition between parasite genotypes might explain the observed pattern leading to selection of these alleles, and thus a boost of homozygote genotypes in the middle of the season. Toward the end of the season, selection appears to relax and we observed a recovery in linkage equilibrium, as well as an increase in effective population size. This might be explained by genetic exchange in these trypanosomes in natural populations.

## Introduction

Colonies of social insects are infected by a wide range of parasites and pathogens. These colonies can be infected by more than one strain of a certain pathogen because the colony conditions facilitate accumulation of pathogens and parasites as the colony members are large in number and form long-lived, warm, and resource-rich nests that provide ideal conditions for the spread of microorganisms (Schmid-Hempel 1998). Indeed, multiple infections have been shown to be quite common in many host–parasite systems (Clark 1977; Day et al. 1992; Read and Taylor 2001; Schmid-Hempel and Reber-Funk 2004). Relative to single infections, infection by several competing parasite genotypes is expected to result in a suboptimal form of host exploitation due to competition among different strains within the host. Thus, multiple infections are predicted to be more virulent than single infections (Levin and Pimentel 2001). However, depending on their relatedness, cooperation between parasite genotypes might occur, resulting in decreased virulence (Schjorring and Koella 2003). Frank (1992) presented an evolutionary stable strategy (ESS) model for the evolution of virulence in which transmission increases with virulence, imposing a trade-off between a parasite's survival and transmission, resulting in a decrease in host recovery with increasing virulence.

As transmission rates are strongly dependent on the availability of susceptible hosts, the host population structure has a strong impact on the transmission rate (Schmid-Hempel et al. 1999). Annual social insect colonies exhibit rapid growth phases after colony foundation by posthibernation queens in spring. Once workers from the first brood emerge, they take over all tasks related to colony maintenance and the queen exclusively engages in oviposition behavior. This division of labor strongly enhances an ergonomic growth phase, that is, rapid growth of the colony over a short period of time. During this time, large numbers of new individuals enter the population serving as potential susceptible hosts, if they have not been previously infected by the queen or other nest mates. This population dynamic greatly enhances the transmission of horizontally transmitted parasites within the colony (Schmid-Hempel 1998). Therefore, throughout the year, it is expected that the transmission rate decreases as more and more individuals become infected, and thus, the number of available susceptible hosts also decreases. But still, the transmission rate becomes higher than the egg-laying rate of the queen during the season. Moreover, the increased rate of intracolonial horizontal parasitic transmission also increases the probability of multiple infections per host (Schmid-Hempel 1998).

Bumblebees are primitively social insects. As one characteristic, they exhibit an annual life cycle. Queens are usually single mated, and hence, all workers are full sisters (Schmid-Hempel and Schmid-Hempel 2000). Once the first workers emerge, colonies enter an ergonomic growth phase during spring and early summer. Depending on the species, colonies vary in the number of workers per colony; for example, *Bombus terrestris* and *Bombus lapidarius* colonies can contain up to 400 workers, whereas those of *Bombus pascuorum*, *Bombus sylvarum*, *Bombus ruderarius*, and *Bombus muscorum* are relatively small (20–100 workers) (Alford 1975). Toward the end of the season, sexuals are produced, mating takes place, and the resultant mated gynes hibernate (Alford 1978).

A broad spectrum of parasites is known to infect bumblebees. One of the most common parasites is *Crithidia bombi*, a trypanosome gut parasite that has several subtle effects on its host (Lipa and Triggiani 1988). Shykoff and Schmid-Hempel (1992) described slower growth of infected colonies during the critical period early in the colony circle and smaller ovary size of infected individuals. Nevertheless, it is highly virulent when residing in hibernated queens, as their success in founding colonies is dramatically reduced, and thus lowering fitness by 40% and more (Brown et al. 2003).

*Crithidia bombi* infections have been studied in detail in both natural populations and ex situ experimental studies. Several tools have been developed for the study of *C. bombi* enhancing its detection and experimental manipulation. Microsatellite markers have been developed (Schmid-Hempel and Reber-Funk 2004) allowing for detailed analyses of genotypes infecting bumblebees. Studies of natural populations have shown that large numbers of distinct genotypes contribute to infections and that multiple infections are quite common (Schmid-Hempel and Reber-Funk 2004; Salathé and Schmid-Hempel 2011). Furthermore, within a colony, individuals are parasitized by more than one strain of this gut parasite (Schmid-Hempel and Reber-Funk 2004). Recently, it has been discovered experimentally that *C. bombi* strains show genetic exchange between genotypes in cases where multiple infections occur (Schmid-Hempel et al. 2011). Moreover, Salathé and Schmid-Hempel (2011) found a striking diversity of infection in several bumblebee hosts in two ecologically different habitats over a time period of 3 years. In addition, their data strongly suggest that a mixture of both sexual and clonal reproduction occurs in natural populations of this trypanosome gut parasite. Also, Erler et al. (2012) suggest, based on their data, that sexual reproduction is an alternative strategy in this parasite. They also detected high levels of multiple infections which might enhance this phenomenon.

Observed prevalences in field populations are typically around 10–30% in Bombus workers within colonies, but this can reach up to 80% in early summer (Shykoff and Schmid-Hempel 1991). Aside from seasonal differences, the genetic composition of the host population might also influence the prevalence of this parasite. In inbred populations or populations with low levels of heterozygosity, the prevalence and abundance of *C. bombi* has been found to be higher than in more diverse populations (Whitehorn et al. 2011).

*Crithidia bombi* can be transmitted horizontally between colonies (intercolonial), when workers forage on flowers already contaminated with infected feces by other workers from other colonies (Durrer and Schmid-Hempel 1994), although workers are able to recognize and avoid contaminated flowers to a certain extent (Fouks and Lattorff 2011). This intercolonial horizontal transmission might enhance the occurrence of multiple infections. Additionally, identical clones of *C. bombi* were found in different host species, also supporting horizontal transmission of this parasite and underpinning the lack of host-specific adaptation (Erler et al. 2012). Furthermore, Ruiz-Gonzáles et al. (2012) stated that the evolution of *C. bombi* to a generalist parasite is due to the following: short-scale specialization (host quality and transmission) is overridden by repeated bottlenecking, combined with the reproductive strategy of this parasite.

In addition, within a single colony, *C. bombi* is passed on among workers and is also transmitted horizontally to the daughter queens (intracolonial), which represent the reproductive offspring. Interestingly, Ulrich et al. (2011) showed that most colonies are capable of filtering a circulating infection before it reaches the young queens and this represents a temporal shift in parasitic composition in the workers. In colonies where workers harbor up to five parasitic strains after artificial infection, the daughter queens were only singly or doubly infected. This study showed a dramatic change in the representation of the five strains used for infection from the day of infection to the production of new daughter queens in the colonies.

We aim to study the impact of temporal changes in host demography on the population structure of a horizontally transmitted parasite. Specifically, along a temporal gradient and in two of the most abundant host bumblebee species (*B. terrestris* and *B. lapidarius*), we investigate (i) the prevalence and intensity of *C. bombi* infection and the corresponding changes throughout the season as the demography of the host population changes along this temporal gradient and (ii) the population genetics of *C. bombi*, determining the frequency distribution of genotypes, the frequency of single versus multiple infections, and the rate of intra- and interspecific transmission.

## Experimental Procedures

### Sampling of bumblebees

Bumblebees were caught during foraging flights in the beginning of June, the middle of July, and in early August 2009 in an urban, flower-rich park, in Halle (Saale), Germany (51° 29′ 27.44″ N; 11° 56′ 11.44″ E). The sampling area was 130 × 130 m. Depending on the sampling date, exclusively workers or workers and drones of *B. terrestris* (June: 27 workers; July: 36 workers, 16 drones; August: 12 workers) and *B. lapidarius* (June: 42 workers; July: 24 workers, 24 drones; August: 66 workers, 6 drones) were collected. Individuals were immediately anesthetized with acetic acid ethyl ester. Bumblebees were determined using the species identification key of Mauss (1994). The intestinal tracts were dissected from the abdomen and the remaining material stored at −20°C for later genotyping.

### DNA extraction and genotyping

The dissected intestinal tracts were homogenized in 500 μL deionised water. Two hundred microliters of the homogenized tissue was transferred to a 96-well microtiter plate and centrifuged for 30 min at 3220*g*. The supernatant was discarded, and the DNA was extracted after standard protocols (Walsh et al. 1991; Popp and Lattorff 2011). For genotyping the bumblebees, a single leg was cut and the same procedure for DNA extraction was conducted as described above. Genotyping and assignment to colonies are necessary to ensure that samples were continuously taken from the same population.

*Crithidia bombi* strains were genotyped using four polymorphic microsatellite loci (Schmid-Hempel and Reber-Funk 2004). Amplification of microsatellites and genotyping were conducted according to Popp and Lattorff (2011). Currently, there is no information, and therefore, it is not known whether these loci are linked to genes which underlie selection (Schmid-Hempel and Reber-Funk 2004).

Bumblebees were genotyped using five highly variable microsatellite markers, originally developed for *B. terrestris* (Estoup et al. 1995, 1996). Multiplex amplification of polymerase chain reaction (PCR) products and genotyping were conducted according to Erler and Lattorff (2010).

### Sibship reconstruction of *Bombus* hosts

In order to assign individual worker and drone bumblebees to colonies, we used the sibship reconstruction algorithm implemented in the software Colony v1.3 (Wang 2004). It is a maximum-likelihood algorithm that takes the population-wide (i.e., sample-wide) allele frequencies into account. All individuals of one of the species were fed into one run of colony. Twenty runs were performed using different random seed numbers. The assignment of individuals to colonies was recorded and from all 20 runs we analyzed the recovery rate of colony assignments. Incorrect assignments were assumed to have a low recovery rate; those assignment groups having a recovery of more than 50% have been chosen for further analysis.

### Prevalence of *Crithidia bombi*

The prevalence of *C. bombi* infections at each sampling date was determined as the relative frequency of infected individuals for each of the two bumblebee species. As sample size was restricted, confidence intervals based on a binomial distribution were calculated using a Java-based tool available at http://statpages.org/confint.html. The differentiation into infections caused by a single genotype or infections caused by multiple genotypes was done on the outcome of genotyping of *C. bombi*. Multiple infections can be definitely assigned when at least one locus has three or more alleles. *Crithidia bombi* is usually diploid, although the ploidy number of the genus *Crithidia* is not generally known. The microsatellite study by Schmid-Hempel and Reber-Funk (2004) strongly indicates that *C. bombi* is diploid, too; however, a single genotype shows a maximum of two alleles when heterozygous. Overall prevalence and the prevalence of multiple infections were analyzed using a generalized linear model (GLM) with a binomial distribution and the logit link function. Species, sampling month, and their interaction were used as fixed explanatory factors.

### Intensity of *Crithidia* infections

In order to quantify the intensity of infection, we used the peak height information of the microsatellites from the *C. bombi* genotyping from all single and multiple infected bumblebees. We assume higher numbers of *C. bombi* cells correspond with increased peak heights, that is, a positive correlation between parasite cells and peak heights. This method is reliable as there is a positive correlation between increasing DNA concentration and the corresponding relative peak heights (Moritz et al. 2003).

Peak heights of all *Crithidia* infections were obtained from genotyping profiles analyzed using MegaBACE Fragment Profiler software (Amersham Biosciences, Freiburg, Germany). All peaks representing single alleles, excluding any stutter bands, were summated per locus and then per locus values summated across all loci. In order to fulfill criteria of normality, data were log-transformed. An analysis of variance (ANOVA) with species, type of infection (single or multiple), and sampling date as fixed factors was used to infer the main factors contributing to the variability in the intensity of infection. Statistical analyses were performed using STATISTICA 6.0 (StatSoft, Tulsa, OK).

### Decomposition of multiple infections into contributing single genotypes

As multiple infections are composed of a combination of different genotypes these are not accessible for population genetic analysis. In order to include them into further analyses on the population structure of *C. bombi*, the individual genotypes contributing to a certain multiple infection need to be reconstructed. An algorithm has been developed by Schmid-Hempel and Reber-Funk (2004), but the approach is not described in sufficient detail to implement in this study.

Here, we develop a new algorithm based on the frequency of alleles within a multiple infection and the relative intensity of the genotypes contributing to a multiple infection. The allele frequency indicated by its relative peak height is composed by the within-genotype frequency and by the between-genotype frequency, the latter being strongly influenced by the ratio of the genotypes to each other. For every multiple-infection allele sizes and their corresponding peak heights were extracted from the capillary sequencer electropherograms. Peak height was assumed to reflect the initial copy number of an allele (B. Fouks and H. M. G. Lattorff, unpubl. data) and was used to calculate the allele frequency within a multiple infection for every locus. Thus, the allele frequency corresponds to the allele specific peak height divided by the sum of all peak heights of that locus. Reconstruction of single genotypes was initiated by randomly choosing one locus for which the lowest frequency (*f*_1_) of an allele was assumed to represent the single occurrence of that allele, present in one of the genotypes only. Depending on the number of distinct alleles occurring at that locus, either a second allele was assigned with a similar frequency (*f*_1_ = *f*_2_) to the same genotype or the other alleles showed a frequency pattern suggesting it belongs to the alternative genotype (1 − *f*_1_). The remaining alleles were assigned to the alternative genotype (*f*_3_; *f*_4_). The ratio of alleles at one locus between the genotypes indicates the frequency distribution of the genotypes (genotype 1 = *f*_1_; genotype 2 = *f*_3_). This decomposition was continued for the remaining loci assuming a similar genotype frequency distribution. The procedure was verified using a different locus as starting point. A representative scheme is given in Figure S2.

### Intra- and interspecific transmission of parasitic genotypes

After decomposition of multiple infections into contributing single-parasite genotypes, we recorded the occurrence of identical parasite strains within and between the two host species across sampling dates. Hence, the number of intra- and interspecific transmission events was detected throughout the season.

## Results

### Sibship reconstruction of *Bombus* hosts

In total, we sampled 93 individuals of *B. terrestris* (both sexes only present in July) and 162 of *B. lapidarius*, where both sexes were present in July and August. These were assigned to 47 and 61 colonies, respectively. Sibship reconstruction, using workers and males, was run 20 times in order to test the rigor of colony assignment. The median recovery for every colony was high with 17 (25% quartile: 13% and 75% quartile: 20) for *B. lapidarius* and 20 (18 and 20) for *B. terrestris*. As the finite sample size influences the observed number of colonies, we used the truncated Poisson method as described in Goulson et al. (2010) to estimate the number of nonsampled colonies. These were 8.12 for *B. terrestris* and 4.95 for *B. lapidarius*. An alternative approach to estimate the number of nonsampled colonies, developed by Cornuet and Aries (1980), was used resulting in 12.1 for *B. terrestris* and 5.8 for *B. lapidarius*. The congruous assignment of individuals to colonies indicated consistent sampling over the season with many colonies being resampled across months and only 10–25% of the colonies were not present in the sample. For *B. lapidarius,* 5, 4, and 14 unique colonies were found for the months June, July, and August, respectively, and four shared colonies recovered in June and July, nine in July and August, and 15 in June and August. Ten colonies contributed individuals in every month sampled. For *B. terrestris*, we found 10, 16, and 6 colonies exclusively for the months June, July, and August. Twelve colonies were recovered in June and July and two in July and August, whereas there were no co-occurring colonies in June and August. One colony was sampled during all 3 months.

The microsatellites used for genotyping showed a high degree of polymorphism and low nondetection errors (Boomsma and Ratnieks 1996): 5.79 × 10^−5^ for *B. lapidarius* and 3.48 × 10^−5^ for *B. terrestris*.

### Prevalence of *Crithidia bombi* infections

**Figure 1:**
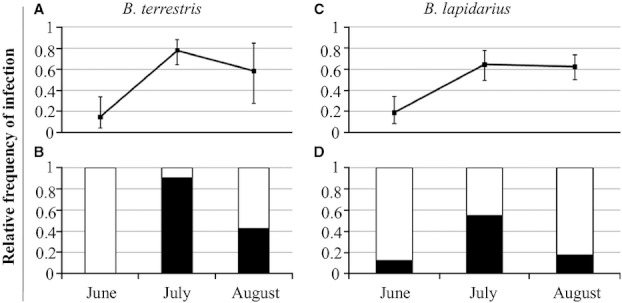
Prevalence of *Crithidia bombi* infection in *Bombus terrestris* (A) and *Bombus lapidarius* (C) individuals between June and August 2009; infections divided into single-infected (white) and multiple-infected (black) for *B. terrestris* (B) and *B. lapidarius* (D). Samples: *B. terrestris* (June: 27 workers; July: 36 workers, 16 drones; August: 12 workers) and *B. lapidarius* (June: 42 workers; July: 24 workers, 24 drones; August: 66 workers, 6 drones). Error bars denote 95% confidence intervals.

For *B. terrestris*, a prevalence of 14.8% was observed in June, increasing to 77.8% in July, and decreasing in August to 58.3% (Fig. 1A). A similar situation was found for the *B. lapidarius* population with prevalences of 19.0% in June, 64.6% in July, and 62.5% in August (Fig. 1C). We used a GLM to test whether the general prevalence of the parasite is affected by the host species, sampling date (month) or by an interaction of both. The GLM showed no significant difference between the two host species for the general parasitic prevalence. In contrast, the infection with *C. bombi* significantly differed between the three sampling events, whereas there was no significant interaction of host species and the three sampling points (Table 1).

**Table 1:** Results of GLM (binomial distribution and logit link function) with backward stepwise removal of nonsignificant factors for the prevalence of *Crithidia bombi* and the distribution of single versus multiple infections including host species and month as fixed factors

Factor	*df*	Wald statistic	*P*-value
Prevalence of *Crithidia bombi*			
Month	2	42.906	**<10**^**−6**^
Single versus multiple infection			
Month	2	29.398	**<10**^**−6**^
Host species × Month	2	12.831	**0.002**

GLM, generalized linear model. Only the final simplified best model is shown. Significant values are shown in bold.

When distinguishing the type of infection into single and multiple infections, we observed that none of the individuals of *B. terrestris* were infected by multiple strains of *C. bombi* in June, whereas in *B. lapidarius*, the rate of multiple infections was 12.5%. The highest prevalence of multiple infections in both species was observed in July (90.5% in *B. terrestris* and 54.8% in *B. lapidarius*). In August, the proportion of multiple infected individuals decreased in both species (42.9% in *B. terrestris* and 17.9% in *B. lapidarius*) (Fig. 1B and D). A GLM was used to test, whether there are differences between species, sampling points and the interaction of these two parameters on the prevalence of multiple infections. Single and multiple infections differed significantly between months, and there was also a significant interaction between host species and month (Table 1). Prevalence did not differ between sexes in months, when both males and females are available (Mann–Whitney *U* test; *B. lapidarius Z* = −1.45, *P* = 0.15 and *B. terrestris Z* = −1.07, *P* = 0.28). For *B. terrestris*, both sexes were available in the samples from July, and for *B. lapidarius*, both sexes were sampled in July and August.

### Intensity of *Crithidia* infections

The intensity of parasitic infection did not differ significantly between species (ANOVA; *df* = 1; *F* = 2.011; *P* = 0.159), or the number of genotypes contributing to an infection (ANOVA; *df* = 1; *F* = 0.629; *P* = 0.429). The intensity of infection did not differ between sexes in either species (ANOVA; *B. terrestris*: *df* = 1; *F* = 0.209; *P* = 0.65; *B. lapidarius*: *df* = 1; *F* = 0,041; *P* = 0.839), although there was an interaction between sex and infection type (ANOVA; *df* = 1; *F* = 5.073; *P* = 0.027) in *B. lapidarius*. Here, singly infected males have a higher intensity of infection than the multiply infected ones, whereas workers show a similar pattern when single and multiple infections are compared. Based on these results, samples were pooled over species, and a significant decrease in the intensity of infection was observed as the season progressed (ANOVA; *df* = 2; *F* = 25.547; *P* < 0.01) (Fig. S1).

### Population genetics of *Crithidia bombi*

In total, we identified 193 parasitic genotypes across the whole season in both the bumblebee host species. Sixty-five genotypes were recorded as single infections and 128 genotypes were found in multiple infections. Among these, 171 genotypes were unique and only 12 were recorded more than once. As the number of observed genotypes was influenced by the finite sample size, we used two different approaches to correct the number of genotypes and estimate the number of nonsampled genotypes. Based on a binomial distribution and assuming an equal distribution for all genotypes, the method by Cornuet and Aries (1980) gave an upper estimate of a total of 777 genotypes. When assuming a Poisson distribution for the genotypes, the zero category of a fitted truncated Poisson distribution indicates the number of nonsampled genotypes. This serves as a lower estimate and results in a total number of 253 genotypes. As the observed distribution does not fit to a Poisson distribution, the expected number of genotypes present in this population is probably closer to the upper estimate.

Using an analysis of molecular variance (AMOVA) (Arlequin v. 3.5.1.2), we tested for the factors influencing the variance in the parasitic genotypes. There was a significant difference of genotypic variance between the host species (*P* = 0.04) and also between the three sampling points (*P* < 0.001), but there were no significant differences between the host individuals within species and the sampling points (*P* = 0.88, Table 2). In addition, the variance of parasitic genotypes between all host individuals did not influence (*P* = 0.31) the overall variance in parasite distribution.

**Table 2:** Results of an AMOVA (weighted average over loci) of *Crithidia bombi* genotypes according to the species they were extracted from (host species, *df* = 2) and sampling date (months, *df* = 3)

	Sum of squares	Variance components	Percentage variation	*P*-value
Among host species	13.250	0.035	2.354	**0.04**
Among months within host species	10.160	0.043	2.851	**<10**^**−5**^
Among individuals within host species and months	236.602	−0.035	−2.333	0.88
Within individuals	265.000	1.452	97.129	0.31
Total	525.012	1.494		

*P*-values were obtained from 1023 permutations. Significant values are shown in bold.

### Genetic diversity within *Crithidia bombi*

**Figure 2:**
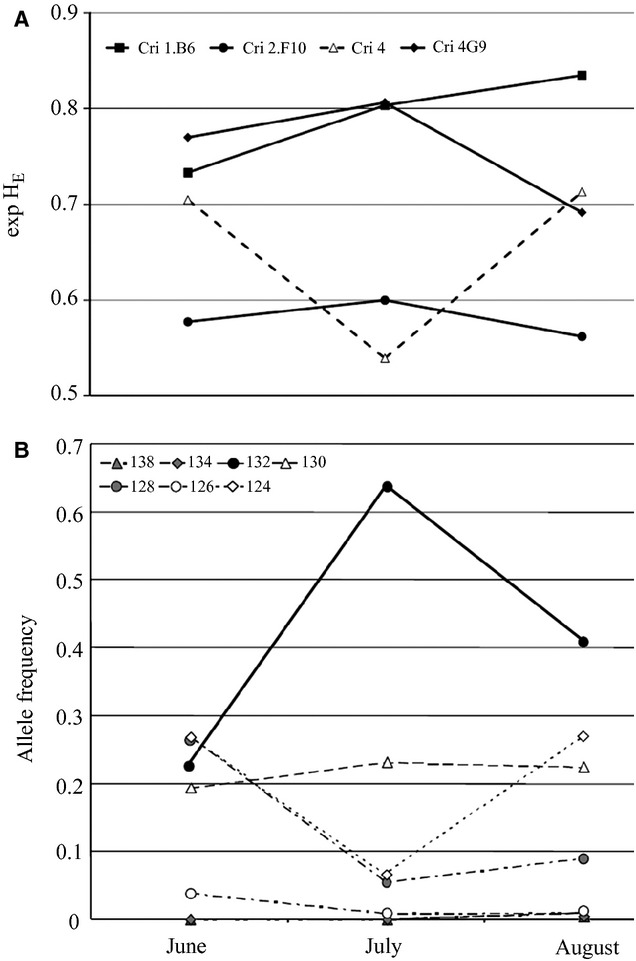
Measurements of the genetic diversity of *Crithidia bombi* throughout the season. (A) Expected heterozygosity for all four microsatellite loci of *C. bombi* and (B) allele frequency distribution of alleles for the microsatellite locus Cri4.

We used both heterozygosity (i.e., the presence of different alleles at one locus averaged over loci) and allelic richness (i.e., the number of alleles per locus corrected for sample size) as measures of the parasite populations' genetic diversity. Factors influencing the heterozygosity of *C. bombi* have been analyzed using an ANOVA with species, locus, and sampling month as fixed factors. Nonsignificant interaction effects have been removed by backward stepwise selection and simplified models tested against more complex models using an ANOVA. The final model included host species as a single factor (*P* = 0.02), with *C. bombi* extracted from *B. lapidarius* having higher levels of heterozygosity (*H*_*E*_: 0.72 vs. 0.67) and a sampling time (month) by locus interaction (*P* = 0.02) (Fig. 2A). The strongest predictor was locus (*P* < 0.001), with two loci (Cri1.B6 and Cri4.G9) showing high levels of heterozygosity (0.79 and 0.76, respectively), whereas the remaining two (Cri2.F10 and Cri4) showed reduced levels of heterozygosity (*H*_*E*_: 0.58 and 0.65). A Tukey's honestly significant difference post hoc test revealed significant differences between each of the high heterozygosity and each of the low heterozygosity loci (Cri1.B6–Cri2.F10: *P* < 0.001; Cri1.B6–Cri4: *P* = 0.002; Cri4.G9–Cri2.F10: *P* < 0.001; Cri4.G9–Cri4: *P* = 0.018). No influence of the sampling time (month) was detected (*P* = 0.86), as the average heterozygosity did not change (June: 0.70, July: 0.69, August: 0.70). However, the locus Cri4 deviated from this pattern, showing a drastic reduction in heterozygosity in July (Fig. 2A). This effect of reduction was more pronounced in *B. terrestris* (*H*_*E*_: 0.45) than in *B. lapidarius* (*H*_*E*_: 0.63), which showed in the other months levels of 0.67 and 0.74, respectively. This drastic decrease in heterozygosity was due to an increase in frequency of a single allele (132 bp) (Fig. 2B), that is virtually absent in June with 26 chromosomal sets being sampled allowing for the detection of alleles with a minimum frequency of 0.04. In July, this allele shows a frequency of 0.64 resulting in 50% of all genotypes being homozygous for 132/132 (Fig. 2B).

This marked increase in frequency of a single allele resulted in strong linkage disequilibrium between all pair-wise comparisons involving Cri4 in July (*P* = 0.003). In contrast, no significant linkage disequilibrium was detected in June or August (Table S1). Selection for the allele 132 in July also led to a reduction in the effective population size in July. Using a point estimate method based on the linkage disequilibrium implemented in LDN_e_ (Araki et al. 2007) values for N_e_ were 63.7, 22.0, and 59.6 for June, July, and August, respectively (Table S2). LDNe is a program with a Visual Basic interface that implements a bias correction for estimates of effective population size (N_e_) based on linkage disequilibrium data.

### Intra- and interspecific transmission of parasitic genotypes (overlap of genotypes)

Our sampling design facilitated the observation of intraspecific (i.e., from one colony to another within one species) and interspecific (from one colony to another between the two host species) *C. bombi* transmission events. One intraspecific and also one interspecific transmission event was observed between June and July, five intraspecific transmissions in July, and three interspecific ones between July and August. One intraspecific transmission of a parasitic genotype occurred in August, and finally, between June and August, there was one genotype of *C. bombi* transmitted intraspecifically. The balanced number of intra- and interspecific transmission events represents additional evidence for *C. bombi* being a multi-host–parasite (Salathé and Schmid-Hempel 2011; Erler et al. 2012).

## Discussion

**Figure 3:**
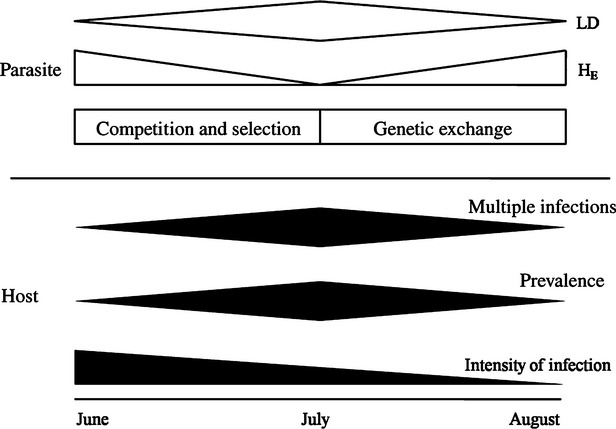
Scheme of the temporal changes in the *Bombus–Crithidia* host–parasite system over a season (June–August).

Our data show that population structure of *C. bombi*, a multi-host–parasite of bumblebees, changed dramatically throughout the year. We observed two distinct phases which might be explained as follows: (1) a phase of competition and selection early in the year, during the ergonomic growth phase of the host and (2) later on in the year, a phase of “relaxation” possibly characterized by the occurrence of genetic exchange (Fig. 3) (Salathé and Schmid-Hempel 2011; Schmid-Hempel et al. 2011). This pattern of infection prevalence being highest during the middle of the season and subsequent decline at the end of the season has been observed in other *Crithida–Bombus* systems, but this is the first study to examine the population structure of both host and parasite (Gillespie 2010; Shykoff and Schmid-Hempel 1991). During the first phase, the prevalence and rate of multiple infection of the parasite increases (Fig. 3), and selection at a gene closely linked to a molecular marker (Cri4) used for the assessment of genetic diversity was detected. This suggests that Cri4 might be involved in competition between clonal lineages of the parasite during multiple infections. This selective pressure leads to a dramatic decrease in the genetic diversity and the effective population size of the parasite population. During the second phase, a large number of new genotypes occur and linkage disequilibrium, which accumulated during the first phase due to the selection for the gene linked to Cri4, breaks down again and the initial genetic diversity of the population is restored (Fig. 3). Intensity of parasite infection continuously declines throughout the year (Fig. 3). This may be due to the enhanced competition between *C. bombi* genotypes during multiple infections, as indicated by similar infection intensities of multiple and single infections, or strain-filtering of the host itself (Ulrich et al. 2011).

In this study, the prevalence and intensity of *C. bombi* infections in local in situ populations of *B. terrestris* and *B. lapidarius* were studied within a year. Similarly, Shykoff and Schmid-Hempel (1991) also investigated local natural populations of bumblebees in Switzerland and their parasite prevalence, including *C. bombi*. The authors' data on the prevalence of *C. bombi* were limited to between late July and August, whereas our data encompass the beginning of summer (June). Although Shykoff and Schmid-Hempel (1991) did not distinguish between sample dates, the observed field prevalence supports our findings concerning prevalence. Furthermore, due to the genetic analysis of the host populations and the high overlap of colonies between the sampling points in our study, differences in prevalence and parasite intensity of infection in our data are not biased as samples were taken from different colonies at the three sampling dates.

The prevalence of *C. bombi* in *B. lapidarius* and *B. terrestris* in this study was 64.6% and 77.8%, respectively. This is comparable to the 80.3% of infected *B. terrestris* reported by Shykoff and Schmid-Hempel (1991) in Switzerland and within the 0–82% range observed by Gillespie (2010) in N. American bumblebees. In addition to the general parasitic prevalence, we also focused on the incidence of infection with multiple strains of *C. bombi*. We detected no multiple infections in *B. terrestris* workers from June. This suggests that, up to this point of time, this host species may not have been exposed to a high frequency of *Crithidia-*contaminated flowers, as colony sizes are relatively small at this early part of the season and the spatial density of infected individuals is low. As a consequence, they do not encounter as many parasitic strains and later transfer it horizontally within their colonies. Correlated with the enormous ergonomic growth phase of the host colonies and the increase in potential contacts between individuals, the relative frequency of multiple-infected bumblebee workers increases, reaching its highest level in July. This increase in multiple infection appears to be caused by a growing number of infected colonies within populations, combined with a greater number of infected workers within each of these colonies that transmit the disease during visits to flowers (Durrer and Schmid-Hempel 1994). Subsequently, the rate of multiple infections then decreases later in August. This may be due to (1) the survival of particular strains due to competition between the strains within the host. Therefore, some strains detected in early summer may become increasingly less frequent during the year. (2) The immune system of the host itself: some strains might be attacked more than others by the immune system resulting in different and temporal variation in expression of antimicrobial peptides (Riddell et al. 2009, 2011). (3) Recombination of parasite strains: although *C. bombi* appears to reproduce primary clonally, Schmid-Hempel et al. (2011) showed that genetic exchange takes place in these trypanosomatids, which are closely related to Leishmania spec, where recombination also occurs (Akopyants et al. 2009). Due to these recombination events, certain new allele combinations have higher competitive abilities compared with their parental allele combinations, resulting in decreased multiple infections. (4) The capability of colonies filtering parasitic strains during the season, so that the parasitic composition within the colonies varies drastically during one season (Ulrich et al. 2011). Strain filtering results in reduction in the number of parasite genotypes, hence lowering the amount of multiple infections. (5) Sampling effects could also be potential causes for lower multiple infection rates later in the season, as we just randomly sampled a small, potentially nonrepresentative, number of individuals. However, sibship reconstruction of the host populations indicates that 80–90% of all present colonies were sampled.

Furthermore, the occurrence of intra- and interspecific transmission events may also explain the reappearance of certain parasitic genotypes within and between the two host species during the year. This was also supported statistically in our data (AMOVA), indicating that there is no difference in parasite genotypic variance between the individuals of the two host species. Additionally, by simple count of reappearances of parasitic genotypes, we found that these shared parasitic genotypes were not present at all three sample dates. Due to one intraspecific and one interspecific transmission event, this was the case for two parasite genotypes present in June and July, but not in August. This could be due to recombination or simply nondetection. In July, three genotypes were the same as in August, but were not detected in June. The reason may be due to sample size limitation and the probability of detecting rare strains when host population size is low. These three parasitic genotypes appeared in both time points, possibly due to interspecific transmission. Thus, both types of transmission occur in nearly equal frequency supporting the low genetic differentiation of the parasite populations between host species.

We did not observe any difference in the intensity of infection with *C. bombi* between species or types of infection, but the intensity of infection did decline over the course of the season despite the increasing density of host bumblebee populations as colony size expands. As competition between the different parasitic strains may occur, the decrease in the intensity of infection could increase selection for certain parasitic genotypes (Schmid-Hempel et al. 1999). Alternatively, Manson et al. (2010) found that the consumption of the nectar alkaloid gelsemine significantly reduces the intensity of *C. bombi* 7 days after infection. Immune priming of the bumblebees might also explain the decrease in intensity later in the season, as infection with *Crithidia* can lead to the expression of immune-related genes in the bumblebee protecting against subsequent infections (Schlüns et al. 2010).

The main factor influencing *C. bombi* genotypic variance is time. As the season progresses, parasite genotypes within the hosts dramatically change from June to August, whereas only slight levels of differentiation were observed between host species. This is also supported by the level of intraspecific versus interspecific transmission. The dramatic loss of parasite heterozygosity in July and the related decline of allelic richness are due to an increase in one allele (132 bp) at the locus Cri4. The molecular marker itself is unlikely to be the target of selection, but it might be tightly linked to a gene that is under selection. As multiple infections of *C. bombi* are highest at that time, this marker may indicate a region that is involved in within-host competition between strains. Negative frequency-dependent selection might be the reason for later depletion of this allele. Yet, another explanation for disappearance of this allele is genetic exchange demonstrated by Schmid-Hempel et al. (2011). As their study is restricted to laboratory conditions, we in turn could show that genetic exchange is one of the indications for sexual reproduction being an alternative reproductive strategy besides clonal reproduction in natural populations, which also has been shown by Salathé and Schmid-Hempel (2011). In summary, our results demonstrate the genetic processes that underlie this complex dynamic system of multi-host–parasite interaction, including host availability and susceptibility, virulence, transmission, and the resulting changes in the population genetic architecture of both the parasite and its host.

The trade-off between transmission and intensity of infection is dynamic and may be highly dependent upon the growth phase of host populations. In annual social insect societies, colonies are found by a single individual, the queen, and then undergo a strong ergonomic growth rate, resulting in a drastic increase in number of workers over a short time period, and therefore, enhancing transmission leading to an increase in the proportion of infected hosts. Our data suggest that high transmission rates result in decreased intensity of infection in *C. bombi*, especially when competition occurs between strains.

## Supporting Information

**Table S1.** Linkage disequilibrium in *Crithidia bombi* populations.

**Table S2.** Effective population size of *Crithidia bombi* populations.

**Figure S1.** Temporal decline in the intensity of infection of the overall parasitic infections (single and multiple infections pooled; bumblebee species pooled) in the season 2009. Error bars denote 95% confidence intervals.

**Figure S2.** Scheme visualizing the decomposition of multiple infections into contributing single genotypes. Peaks indicate the different alleles at each locus.
