# Supplementary material for: Seasonal variability of prevalence and occurrence of multiple infections shape the population structure of Crithidia bombi, an intestinal parasite of bumblebees (Bombus spp.)
Source: Microbiologyopen. 2012 Sep 23;1(4):362–72. doi: 10.1002/mbo3.35 (PMC3535382; doi:10.1002/mbo3.35)
Supplement: Supplementary file 1 [file mbo30001-0362-SD3.doc]

**Seasonal variability of prevalence and occurrence of multiple infections shapes the population structure of *Crithidia bombi*, an intestinal parasite of bumblebees (*Bombus spp.)***

Mario Popp, Silvio Erler, H. Michael G. Lattorff

Supplementary material

**Table S1**. Linkage disequilibrium in *C. bombi* populations.

|  | **June** | **July** | **August** |
| --- | --- | --- | --- |
| **Cri4 x Cri2.F10** | 0.619 | **0.003*** | 0.078 |
| **Cri4 x Cri1.B6** | 1.000 | **0.003*** | 0.061 |
| **Cri4 x Cri4.G9** | 1.000 | **0.003*** | 0.067 |
| **Cri1.B6 x Cri4.G9** | 0.447 | 0.006 | 0.236 |
| **Cri1.B6 x Cri2.F10** | 0.619 | **0.003*** | 0.078 |
| **Cri2.F10 x Cri4.G9** | 0.278 | **0.003*** | 0.464 |

p-value of nominal 0.05 level adjusted by 360 permutations to p = 0.003; significant values are marked in bold and with an asterisk

**Table S2**. Effective population size of *C. bombi* populations.

|  |  | **June** | **July** | **August** |
| --- | --- | --- | --- | --- |
| **LAF = 0.05** | Ne | 22.3 | 13.8 | 50.6 |
|  | 95% C.I. | 5.2 – inf. | 6.5 – 25.2 | 19.4 – inf. |
| **LAF = 0.02** | Ne | 63.7 | 22.0 | 59.6 |
|  | 95% C.I. | 21.5 – inf. | 12.6 – 37.9 | 15.7 – inf. |
| **LAF = 0.01** | Ne | 63.7 | 26.3 | 155.9 |
|  | 95% C.I. | 21.3 – inf. | 15.5 – 45.3 | 17.7 – inf. |

LAF: lowest allele frequency used; 95% C.I.: 95% confidence interval determined by jackknifing over loci; inf.: infinity


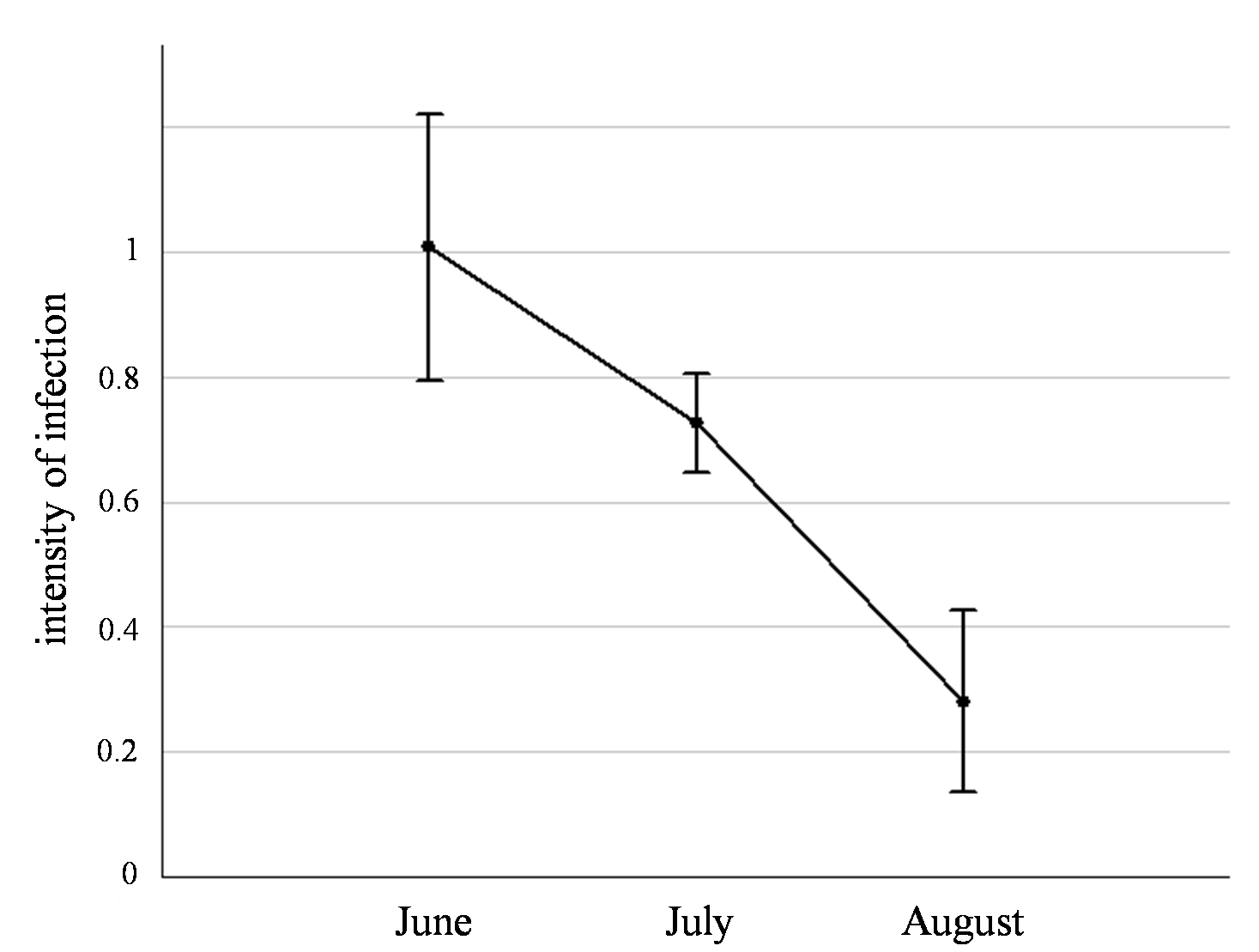


**Fig. S1** Temporal decline in the intensity of infection of the overall parasitic infections (single- and multiple infections pooled; bumblebee species pooled) in the season 2009. Error bars denote 95% confidence intervals.


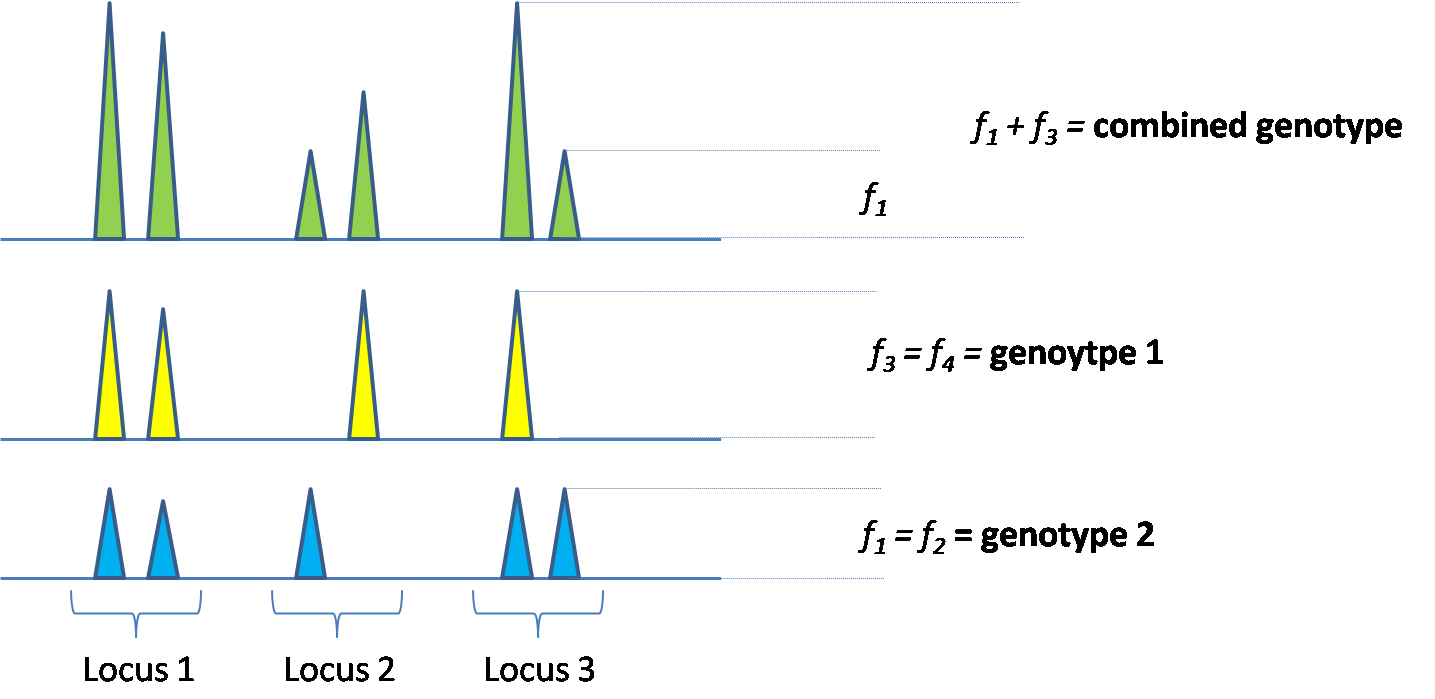


**Fig. S2** Scheme visualising the decomposition of multiple infections into contributing single genotypes. Peaks indicate the different alleles at each locus.
