# Supplementary figures and images for: Seasonal variability of prevalence and occurrence of multiple infections shape the population structure of Crithidia bombi, an intestinal parasite of bumblebees (Bombus spp.)
Source: Microbiologyopen. 2012 Sep 23;1(4):362–72. doi: 10.1002/mbo3.35 (PMC3535382; doi:10.1002/mbo3.35)

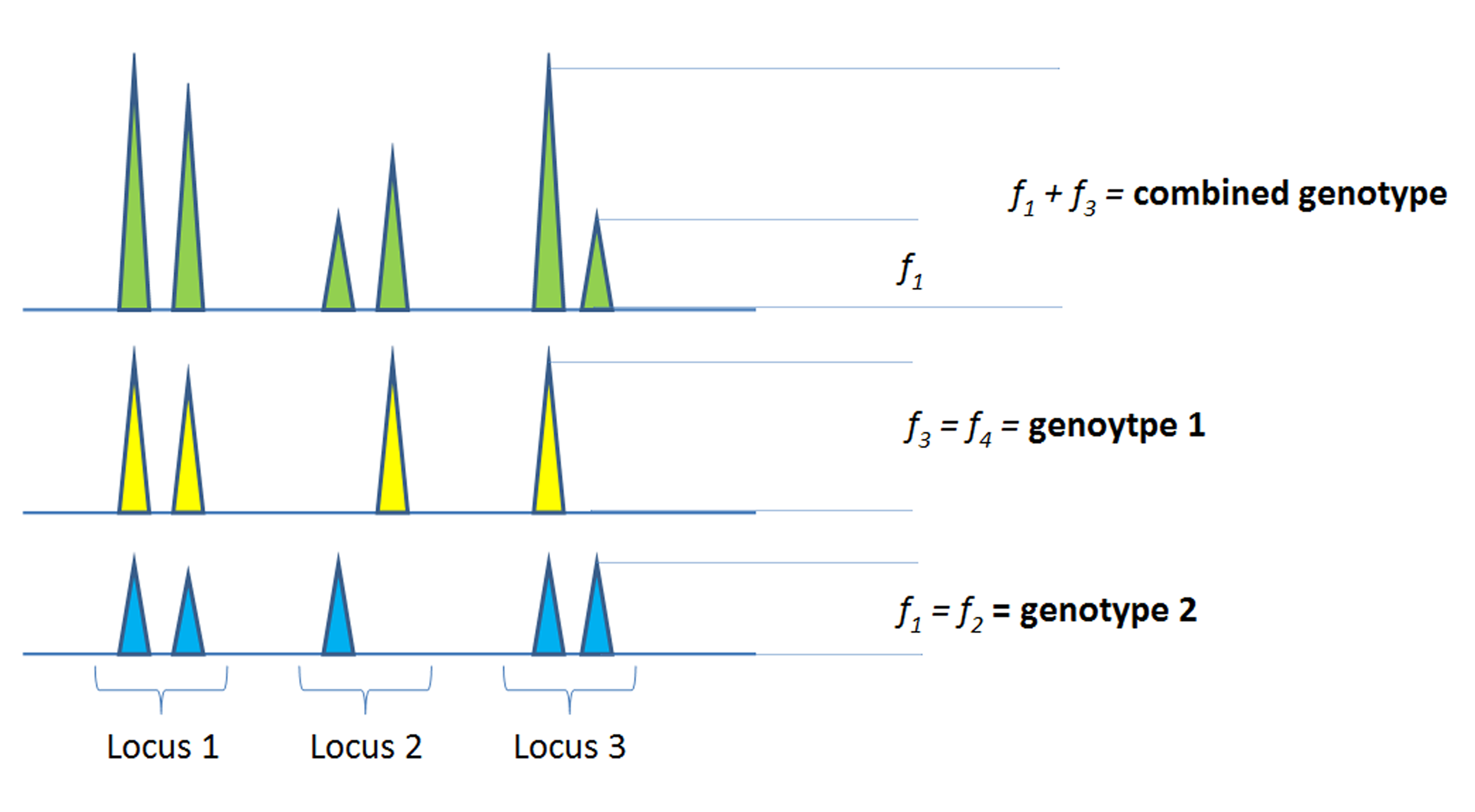

Supplement: Supplementary file 4 [file mbo30001-0362-SD2.tif]
